# Evaluating the Performance of Hybrid Capture 2 Test as a Primary Screening Test from Studies Conducted in Low and Middle-Income Country Settings- Special Focus India

**DOI:** 10.31557/APJCP.2021.22.8.2709

**Published:** 2021-08

**Authors:** Kavita V Anand, Sharmila A Pimple, Atanu Bhattacharjee, Gauravi A Mishra, Surendra S Shastri

**Affiliations:** 1 *Homi Bhabha National Institute, Mumbai. India. *; 2 *Department of Preventive Oncology, Tata Memorial Centre, Mumbai. India. *; 3 *Department of Biostatistics, Centre for Cancer Epidemiology, Tata Memorial Centre. Mumbai. India. *; 4 *Department of Preventive Oncology, Tata Memorial Centre, Mumbai, India. *; 5 *Department of Health Disparities Research, Division of Cancer Prevention & Population Science, M.D Anderson Cancer Centre, Houston,USA. *

**Keywords:** Human papilloma virus, HC2 test, HPV, cervical cancer screening, sexually transmitted infections

## Abstract

Among the screening tests for cervical cancer, advantages of screening with second generation molecular Hybrid Capture 2 (HC2) test is the high sensitivity and negative predictive value that makes it easy to implement as a cervical cancer screening policy necessitating less screening rounds. High income countries are now implementing HC2 test in their national cervical cancer screening program. Since the acceptance of any screening test depends on the sensitivity of the test, the current study was carried out to evaluate the sensitivity of HC2 test reported from Low- and Middle-income countries (LMIC) which share major burden of cervical cancer globally and to establish if HC2 test could be used as a primary screening test in India.

**Materials and methods:**

The population based cross sectional studies from LMICs which evaluated HC2 test as a primary screening modality to diagnose Cervical intraepithelial neoplasm grade 2 and above (CIN2+) lesions were included.

**Results:**

A total of 18 studies from LMIC involving 1,13,086 women were reviewed for sensitivity of HC2 as a primary screening test. The overall average sensitivity and specificity to diagnose CIN2+ lesions were 79.84% (95% CI-71.01,86.73) and 85.63% (95% CI- 84.37,86.92) respectively. India demonstrated an average sensitivity and specificity of 65% (95% CI 57,77) and 93% (95% CI- 92,94) respectively.

**Conclusion:**

Results from LMIC demonstrate a comparably low sensitivity of HC2 test to diagnose CIN2+ lesions as compared to that reported from High income countries. Sensitivity of HC2 was substantially low for India. The current study discusses issues of HC2 assay and the role of untreated Reproductive tract infections as probable causes for low sensitivity of the test. This needs further research in an attempt to improve the sensitivity of the test in an era of self-sampling and low-cost HPV test on horizon to improve the coverage for cervical cancer.

## Introduction

The epidemiological studies have demonstrated the causal role of persistent oncogenic Human papillomavirus (HPV) infection as a main cause for cervical cancer (Walboomers et al., 1999; Bosch et al., 2002; IARC working group., 2007). Various screening tests aid in the diagnosis of precancerous stage of cervical cancer. The cytology-based screening demonstrated significant decrease in cervical cancer mortality rates for developed countries (High income countries) but similar results were not evident for Low- and middle-income countries (LMICs). The cytology-based screening has limitation in terms of sampling technique, transportation issues, interpretation skills of laboratory personnel (Lazcano-Ponce et al., 1999; WHO, 2006), and short screening intervals needed due to low sensitivity of the test reported (Aggarwal et al., 2010). Screening with Visual inspection with 5% acetic acid (VIA) is a proven feasible and cost-effective test for LMICs at present but it poses limitation in terms of the cost and time factors in training the health care providers, subjective biases in interpretation of test results and the burden on tertiary care centers due to its issues of false positive test results (Sankaranarayanan et al., 2003; Gaffikin et al., 2003; Shastri et al., 2014). The direct detection of HPV in cervical specimen by a molecular test offers an alternative for cytology/ VIA based screening. 

Among the molecular HPV tests available, the second-generation molecular Hybrid capture 2 tests (HC2, Qiagen, Gaithersburg), is a extensively validated test across different settings and identifies women at risk of developing cervical cancer, needing a close follow up. The test has probes for all 13 recognised oncogenic HPV types, reported worldwide (Malloy et al., 2000; Qiagen, 2008; Bruni et al., 2010). It becomes easier to implement HPV based screening program due to its advantages (WHO, 2006; Quigen, 2008) - high sensitivity reported from High income countries, safe longer screening interval if tested negative, minimal manpower for performing the test, the potential of self-collection, the reproducibility and standardization of the test with no inter and intra subjective variability. 

HPV testing is now a recommended screening test for cervical cancer (IARC, 2005). From emerging evidences, the High-income countries have now shifted from cytology to molecular HC2 based screening in their national screening programs for cervical cancer (Malloy et al., 2000; Cuzick et al., 2008; Arbyn et al., 2010) while LMICs are still contemplating on using HPV test for cervical cancer screening for their country. There are encouraging results from well conducted population-based trials from LMICs like India (Sankaranarayanan et al., 2009) and Mexico (Flores et al., 2003) demonstrating that, a simple and reliable molecular HC2 test which is now available even in low-income countries has a potential to be accepted as primary screening tool in the near future. 

Since the potential of any screening test to be adopted by a country depends on the sensitivity and specificity (test characteristics), the aim of current review was to appraise the test characteristics of HC2 as a primary cervical cancer screening test reported from LMICs, especially from Indian context. 

## Materials and Methods

*Inclusion criteria*

1] Population-based, cross-sectional studies from LMICs which evaluated the test characteristics of second generation of HC2 test as a primary screening test to diagnose high grade precancerous lesion of cervix on histopathology (CIN2+ lesion). 2] The cervical samples collected by Physician/ trained health care workers. 3] The studies using the value of 1 and above Relative light unit (RLU) corresponding to 5000 or more viral copies per ml as a cut off ratios for interpretation of a positive test results. 4] The gold standard verification was either colposcopy or colposcopy directed biopsy to diagnose severity of the diseases. 

*Exclusion criteria*

1] Studies providing data on test characteristics of HC2 as a triage test, on self-collected cervical samples or among high-risk population (HIV or Sex workers or any other immunocompromised population). 2] Studies using other molecular HPV test including signal amplification test (Care HPV) as a primary screening test as these tests are currently limited to research settings. 3] The studies using first-generation Hybrid capture test (HC1) since the test lacks all the probes of recognised oncogenic HPV till date. 4] Studies not reporting respective Confidence Intervals (CI) for the reported test characteristics of HC2 assay. 5] Studies reporting the test characteristics of HC2 test using cytology as a gold standard. 6] Hospital based studies, as women are expected to be high risk for precancerous or cancerous lesions and may not reflect the true scenario.

*Data sources and searches*

This paper is based on information gathered from published peer-reviewed articles on cervical cancer screening in PubMed data base from year 2000 to 2019 since majority of articles on molecular HPV testing were published after year 2000. The keywords and their corresponding MeSH term used to build up search strategy were ‘cervical cancer screening’ OR ‘cervical intraepithelial neoplasm’ OR ‘cervical dysplasia’ AND ‘human Papillomavirus’ OR ‘HPV test’ OR ‘HC2 test’ NOT ‘HPV vaccination’ NOT ‘HPV self-sampling’ We examined bibliographies of relevant articles to identify additional references. Journal articles fulfilling the inclusion criteria were included in the study. 

**Figure 1:**
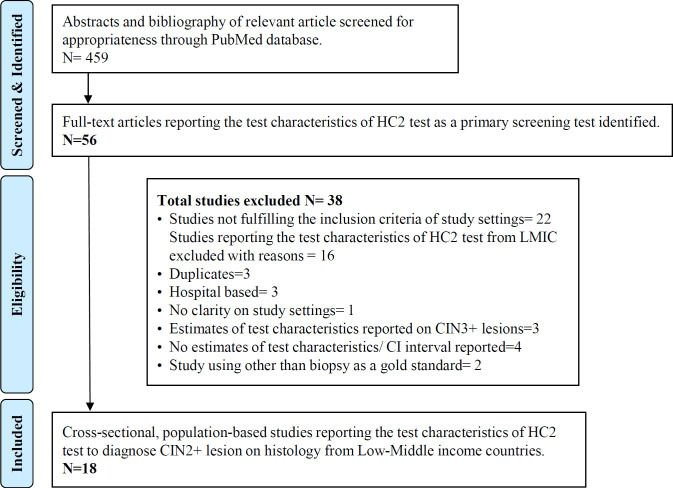
Search Flow Chart. LMICs, Low- and Middle-income countries

Figure 1 - The search strategy identified 459 articles which were screened for appropriateness to the study topic. The bibliography of relevant article was screened for additional references. A total of 56 studies were identified that used molecular HC2 test (Qiagen) as a primary screening test for cervical cancer. These studies reported the test characteristics of HC2 as a standalone test AND as a comparator test to other recognized screening modalities for cervical cancer and other molecular HPV tests. These articles were then reviewed for the study settings. Total of 38 studies were excluded with reasons - 22 studies were clearly excluded as articles did not fulfill the inclusion criteria for study settings. 16 studies from LMICs were excluded with reasons: Studies reported test characteristic of HC2 on hospital based data (Sodhani et al., 2006; Ma et al., 2010; Bhatla et al., 2012), study had no clarity on type of settings (Katyal et al.,2011), studies reported estimates of HC2 on CIN3+ lesion only (Moy et al., 2010; Belinson et al., 2011; Nieves et al., 2013), studies mentioned no estimates of test characteristics of HC2 test or CI intervals (Schiffman et al., 2000; Sankaranarayanan et al., 2009; Cagle et al., 2010; Li et al., 2010; Girianelli et al., 2006), duplicate data, cytology as gold standard (Belinson et al., 2001; Blumenthal et al., 2001; Longatto-Filho et al., 2012; Katanga et al., 2019). Total 18 population based cross-sectional studies fulfilling the eligibility criteria were included in the analysis. 

*Statistical analysis*

The published test characteristic of HC2 as a primary screening test from the LMIC were used to calculate weightage average sensitivity and specificity of the HC2 test. Separate analysis was done to demonstrate the test characteristics of HC2 from Indian context as a primary focus of the study. The weightage average of sensitivity, specificity, 95% CI of sensitivity and specificity were analyzed and forest plots were constructed using R software. 

## Results

Table 1: The Table summarizes characteristics of population based, cross-sectional studies from LMICs, which evaluated second generation HC2 test as a primary cervical cancer screening modality to diagnose CIN2+ lesion. A total of 18 studies from LMIC involving 113086 women were reviewed for test characteristics of HC2 as a primary screening test. The overall weightage average sensitivity of 79.84% (95% CI-71.01,86.73) and specificity of 85.63% (95% CI- 84.37,86.92) to diagnose CIN2+ lesions was demonstrated for LMIC. There are limited population based cross- sectional studies from low-income countries which evaluated the performance of HC2 test as a primary screening test. The calculated average sensitivity and specificity reported for the countries was 82.69% (95% CI-72.13,90.8) and 74.81% (95% CI- 72.01,77.04). Among the Middle-income countries, majority of studies are from India and China. Within the Middle-income countries, there is a significant difference in sensitivity of HC2 test demonstrated between Upper and Lower middle-income countries. The upper middle-income countries demonstrated a high accuracy of HC2 to diagnose CIN2+ lesion, 91.18% (95% CI- 85.07,95.06) as compared to 65.66% (95% CI- 56.01,74.34) demonstrated for lower middle-income countries, while specificity reported were 86.41% (95% CI- 85.86,87.71) and 95.66% (CI-95.23,95.99) respectively. 

**Figure 2:**
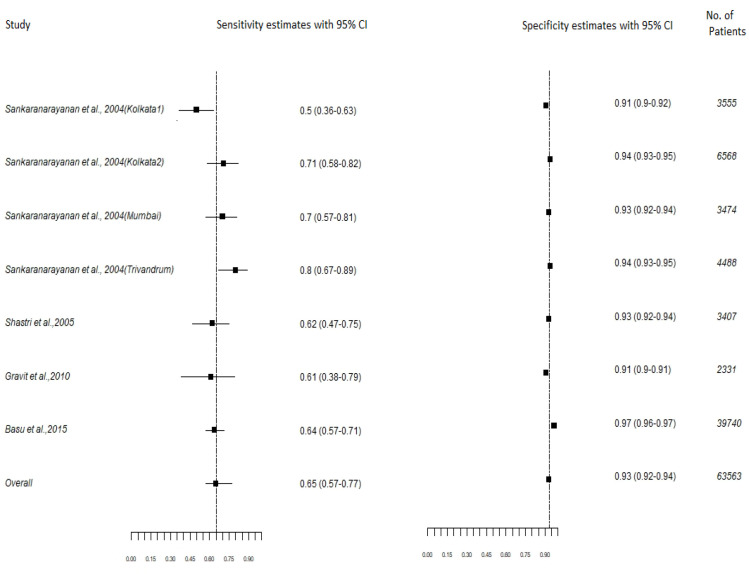
Population Based Cross-Sectional Studies from India Reporting the Test Characteristics of HC2 Test as a Primary Screening Test to Diagnose CIN2+ Lesions

Figure 2: A total of 7 cross-sectional, population-based studies from India involving 63,563 women were reviewed for test characteristics of HC2 test. The age of the women ranged from 25- 60 years, where increase chances of persistence HPV infection is demonstrated. The average weighted sensitivity and specificity demonstrated was 65% (95% CI- 57,77) and 93% (95% CI- 92,94) respectively.

**Table 1:** Summary of Studies from Low- and Middle-Income Countries that Reported the Test Characteristic of HC2 Test as a Primary Screening Test to Diagnose CIN2+ Lesions

	Country	Age of women	Population size	Sensitivity (95% CI)	Specificity (95% CI)	Ref
Low-income countries						
1	Zimbabwe	25-55	2140	81% (75-86)	62% (59-64)	Womack.,2000
2	South Africa	35-65	424	88.4 % (76.9-81.9)	81.9% (76.5-86.5)	Kuhn.,2000
3	Democratic republic of congo	30& above	1528	83.4% (66.8-100)	90.8% (89.0-92.7)	Mahmud.,2011
	Average ^Ŧ^			82.69% (72.13-90.8)	74.81% (72.01-77.04)	
Low middle-income countries						
4	India*	25-65	3555	50% (36.6-63.4)	91.7% (90.7-92.6)	Sankaranarayanan et al.,2004
	India*	25-65	6568	71.7%(58.6-82.6)	94.5% (93.9-95.0)	Sankaranarayanan et al.,2004
	India**	25-65	3474	70.5% (57.4-81.5)	93.6% (92.7-94.4)	Sankaranarayanan et al.,2004
	India***	25-65	4488	80% (67.7-89.2)	94.6% (93.9-95.3)	Sankaranarayanan et al.,2004
5	India	30-65	3407	62 % (47.2-75.4)	93.5% (92.6-94.3)	Shastri et al.,2005
6	Vanuatu	30-50	514	81% (61-93)	94% (91-95)	McAdam et al.,2010
7	India	25-60	2331	61.2% (38.5-79.95)	91.0% (90.5-91.5)	Gravitt et al.,2010
8	India	30-60	39,740	64.4% (57.6-71.0)	97% (96.8-97.1)	Basu et al.,2015
	Average ^Ŧ^			65.66% (56.01-74.34)	95.66% (95.23-95.99)	
Upper middle-income countries						
9	Mexico	15-85	7868	90.7% (83.4-95.0)	93.2% (92.1-93.3)	Salmeron etal.,2003
10	China	35-45	1836	95.2% (88.1-98.7)	85.9% (84.1-87.5)	Pan et al.,2003
11	China	35-50	8497	96.8% (95.0-98.6)	79.7% (78.9-80.5)	Belinson et al.,2003
12	Latin America	18-60	4195	82.8% (76.3-88.4)	86.4% (85.3-87.5)	Sarian et al.,2005
13	Peru	25-49	5435	77.3% (70.4-83.5)	89.3% (88.5-90.1)	Almonte.,2007
14	China	30-54	2388	97.1% (93.2-100.0)	85.6% (84.2-87.1)	Qiao et al.,2008
15	China	15-59	2562	90.4% (83.3-94.7)	86.4% (85.0-87.7)	Li et al.,2009
16	China	25-59	2090	88.9% (70.8-97.6)	84.5% (82.8-86.1)	Wu et al.,2010
17	China	16-54	2625	97.9% (88.7-100)	90.2% (90.0-91.3)	Belison et al.,2012
18	China	25-65	7421	95.8% (91.2-98.5)	87.1% (86.3-87.9)	Zhao et al.,2013
	Average^ Ŧ^			91.18% (85.07-95.06)	86.41% (85.86-87.71)	
	Grand average^Ŧ^			79.84% (71.07-86.73)	85.63%(84.37-86.92)	

^Ŧ^, Weighted average; Among the studies included, 1 Indian multicentric study reported the test characteristics of HC2 test across 4 different centres in 3 locations, Chittaranjan National Cancer Centre, Kolkata 1,2*, Tata Memorial Hospital; Mumbai**, Regional Cancer Centre; Trivandrum***

## Discussion

The current study focused on HC2 (Qiagen) as a primary screening test as the test is widely evaluated and a recommended test for cervical cancer screening. Since the primary focus of any screening test is to diagnose the lesion at early stage, we analysed data using only CIN2+ lesions as endpoint as it provides more time frame to treat patients effectively if diagnosed than CIN3+ lesions particularly in resource constrain countries. There is a difference in sensitivity demonstrated in the studies from LMICs and those reported from High income countries. 

The studies from Europe and America which evaluated the HC2 test as a primary screening test reported sensitivity and specificity estimates of 97.9% (95% CI- 95.9%- 99.9%) and 91.3% (95% CI- 89.5 % -93.1%) respectively for HC2 test to diagnose CIN2+ lesions (Arbyn et al., 2006). The similar findings of high sensitivity (96%) of HC2 assay were also reported from these countries by Arbyn M et al in their meta-analysis (Arbyn et al., 2012). These results support HC2 test as a primary screening test for cervical cancer.

The above findings are inconsistent with the results demonstrated from LMICs. The overall average sensitivity and specificity demonstrated for the test was 79.84% and 85.63% respectively (Table 1). Among the LMICs, the test was widely evaluated in China and India. Studies from China report the test characteristics comparable to High income countries. The probable reasons for low sensitivity of HC2 test reported from other LMICs countries are – improper sampling techniques (Sankaranarayanan et al., 2004) degradation of HPVDNA due to temperature fluctuations during transportation and storage (Almonte, 2007) prevalence of HPV in a geographical area, RLU/CO cut off recommended for interpretation of positive test results for respective countries (Womack et al., 2000) and prevalence of HPV type in a particular geographical area not included in probe cocktail of HC2 assay (Arbyn et al., 2008). Among the data of sensitivity of HC2 test presented in the current review, though South Africa (Kuhn et al., 2000) reported a good sensitivity of 88.4%, the main limitations of this study were verification biases, small retrospective sub- grouped sample tested with HC2 test. 

The studies from India were evaluated separately for test characteristics of HC2 test as a primary screening test (Figure 2). These studies demonstrated homogeneity for age as they enrolled women in age group of 25-65 years where persistence of HPV infection is demonstrated. All studies accounted for verification bias, as all women enrolled in the study underwent colposcopy and biopsy were taken for cervical abnormalities except one study from Kolkata which reported the test characteristics after adjusting for verification biases (Basu et al., 2015) The average sensitivity and specificity of HC2 test demonstrated for India was 65% (95% CI- 57, 77) and 93% (95% CI-92, 94) respectively which is substantially very low as compared to other countries.

A study conducted by Bruni et al., (2010) on a million women with normal cytological findings across the world to detect prevalence of cervical HPV by molecular HPVDNA test, the author mentioned, though the prevalence rate of cervical HPV were lower for India, the country reports high incidence rates of cervical cancer. This comment suggests a possibility of missing HPV infections by molecular HPV test leading to false negative test results. According to the published literature (Burd et al., 2003) the false negative test results of HC2 test is estimated to be 7.5%. Interference of contraceptive jelly, vaginal pessaries, vaginal douches are reported to hamper the diagnostic performance of the tests. Though HC2 test is the most validated test across various settings, main limitation of the test is lack of inbuilt mechanism to monitor cell adequacy. The assay needs 5000 viral copies in a cervical sample to be interpreted as a positive test. The test result ‘HPV not detected’ opens up a debate, whether the cervical sample is truly negative for HPV or the viral load is less than the detection ratio of the test assay due to less cells collected (Malloy at al., 2000; Jastania et al., 2006). There has always been a concern about blood and mucous associated with reproductive tract infections (RTIs) interfering with the sensitivity of screening test for cervical cancer (Sasieni et al., 2003; Sankaranarayanan et al., 2004). The concomitant STIs affecting the test performance of HPV testing has also been commented (Cuzick et al., 2006). A prospective study (Liu et al., 2013) which estimated the prevalence of HPV genotype among three groups of women; group 1-women with mucopurulent cervicitis, group 2- healthy women with no cervicitis and group 3- women with Invasive cancer, the author reported 10% higher failure rates to extract cervical HPV DNA among women with cervicitis. This finding demonstrates the potential of HPVDNA test to be influenced by cervicitis caused by RTIs. 

India reports a huge burden of undiagnosed and untreated RTIs infections (Durai et al., 2019). The prevalence is expected to be high due to illiteracy, ignorance, social stigma, cultural norms, lack of quality health care facilities for screening and treating RTIs and poor access to health care facilities, if available (Garg et al., 2001; Prasad et al., 2005; Ray et al., 2008). At present there are gaps in literature regarding the burden of RTIs induced cervicitis among Indian population. To our knowledge, the only study reported the prevalence of cervicitis to be 55% in Indian population (Tribhovandas et al., 2013). The RTIs caused by bacterial microbes and the genital HPV infections are common among women in reproductive age group and both infections are sexually transmitted. Literature supports, cervicitis (inflammation) associated with RTIs to serves as a co-factor for acquisition and persistence of HPV infection among women. Inflammation modulates the progression of HPV infection to precancerous and cancerous lesion of cervix (Castle et al., 2003; Woodman et al., 2007; Williams et al., 2011). These group of women with concomitant infections (RTIs and HPV) needs prompt detection of HPV along effective treatment of RTIs. 

With the prospect of using low-cost HPV test as a primary screening test in near future the data of HC2 as a primary screening test from India is warned. In the present review the population based cross- sectional studies from India reported a substantial low estimated of sensitivity of HC2 test. We assume that, RTIs among women resulting in mucopurulent discharge are likely to hamper the detection rates of cervical HPV in women with concomitant infection due to cell inadequacy. This may lead to less viral copies below the detection threshold of HC2 test assay resulting in false negative test results which has a potential to affect the sensitivity of the test. At a country level, literature supports HPV based screening program, primarily due to benefits of preventing the repetitive screening costs which can burden the already strained health budget of LMIC. With ongoing research on molecular HPV assay on self-sampling modalities in anticipation to increase the coverage of cervical cancer screening and introduction of care HPV test (Qiagen) that can be conducted on field which are supposed to be low cost, adds to the advantages of screening strategy based on molecular HPV testing as a stand-alone test. Considering the results of the present study and above discussion centered to specific issues with HC2 assay, it becomes relevant to evaluate the effect of mucopurulent discharge due to RTIs on HC2 assay. If the suggested hypothesis is proven, then it would have an important public health implication of treating cervicitis before performing HC2 test in an attempt to reduce the false negative test results. 

## Author Contribution Statement

None.
